# The Effects of Combined Infill Patterns on Mechanical Properties in FDM Process

**DOI:** 10.3390/polym12122792

**Published:** 2020-11-26

**Authors:** Mohammadreza Lalegani Dezaki, Mohd Khairol Anuar Mohd Ariffin

**Affiliations:** Department of Mechanical and Manufacturing Engineering, Universiti Putra Malaysia, Serdang 43400, Malaysia

**Keywords:** additive manufacturing, polylactic acid, fused deposition modeling, polymer, 3D printing

## Abstract

Fused deposition modeling (FDM) is commonly used to print different products with highly complex features. Process parameters for FDM are divided into controllable or uncontrollable parameters. The most critical ones are built orientation, layer thickness, infill pattern, infill density, and nozzle diameter. This study investigates the effects of combined infill patterns in 3D printed products. Five patterns (solid, honeycomb, wiggle, grid, and rectilinear) were combined in samples to analyze their effects on mechanical properties for tensile strength analysis. Polylactic acid (PLA) samples were printed in different build orientations through two directions: flat and on-edge. The limitation was that the software and machine could not combine the infill patterns. Thus, the patterns were designed and assembled in computer aided design (CAD) software. Finite element analysis (FEA) was used to determine the patterns’ features and results showed honeycomb and grid have the highest strength while their weights were lighter compared to solid. Moreover, 0° samples in both flat and on-edge direction had the strongest layer adhesion and the best quality. In contrast, perpendicular samples like 60° and 75° showed poor adhesion and were the weakest specimens in both flat and on-edge, respectively. In brief, by increasing the build orientation, the strength decreases in this study.

## 1. Introduction

These days, developing complex products is achievable thanks to the additive manufacturing (AM) due to flexibility and freeform design. AM opens new ways to build and produce products that traditional processes are not able to meet the dimensional accuracy. Cost reduction, productivity, short processing time, customized products, and faster production lines are advantages that AM brings to industries. 3D printing (3DP) is an alternate name for AM process which was used for rapid prototyping techniques. It can be used in automotive industries [1], medical applications [2], aerospace [3], and construction [4]. Over the past few decades, there have been competitions to increase productivity and quality without sacrificing the products among industries. Thus, AM is a potential option to help manufacturing companies to meet the demand. In novel researches. AM processes have been chosen as a capable process to print soft and smart materials, which is known as 4D printing, as well [5]. This process is classified into Vatphotopolymerization [6], Material extrusion [7], Material jetting [8], Powder bed fusion [9], Sheet lamination [10], Binder jetting [11], and Direct energy deposition [12,13]. Solid, liquid, and powder are the main materials that are used in this process depend on process characterization. From metal alloys to plastics have been used in these processes based on needs and requirements. Polymers, composites-based polymers, hybrid polymers, and thermosets are the most common materials due to their features and ease of adoption. However, the range of material is limited for these processes and more materials should be analyzed to become more compatible. The nature of this technology is to build the products layer by layer from bottom to the top which is in contrast with conventional processes. As an example, in the computer numerical control (CNC) process [14], the machine starts to remove excess material from a bulk to reach the desired shape while AM produces a product by melting or sintering the material in each layer and it repeats it until the sample is completed. The first step is to slice the design by exclusive software and convert it to Stereolithography (STL) format, which is readable by AM machines. Then, the machine follows generated G-codes to print each layer and bind layer together in melting temperature [15]. Ariffin et al. [16] made a comparison between CuraEngine^®^ and Slic3er^®^ software to analyze surface texture and mechanical properties of 3DP specimens. This means each software has unique features that affect dimensional accuracy and surface integrity. Another advantage of AM is producing products without tooling which helps to eliminate friction and collision. By removing these issues, there are no concern about cracking sample during machining process. In these processes, the machine must use support structure to print a layer based on different printing angles.

This support structure can be from the same material or different materials. However, support affects surface texture and after removing the support structure, surface quality may not be as good as other faces. In spite of AM is a powerful technology to build products, anisotropic behavior, and sensitivity of process parameters play an important role as well. These elements are divided into different features based on machine specifications. Finding proper parameters is vital to enhance surface quality and mechanical properties [17]. Therefore, reaching high accuracy and properties are achievable by manipulating critical parameters’ values. There is a wide range of factors but common parameters among all technologies are layer thickness, build orientation, infill pattern, and temperature [18]. Moreover, factors like humidity, material properties, and environment have also effects on the printing process. Unfortunately, controlling these factors is difficult and they may change the mechanical properties of final products. Besides, there are other limitations like staircase defects in AM that highly effective on surface texture [19]. Many technologies are involved in AM and have various features but one of the most common process which is widely used in domestic and industries is Fused deposition modeling (FDM) or material extrusion [20].

**Figure 1:**
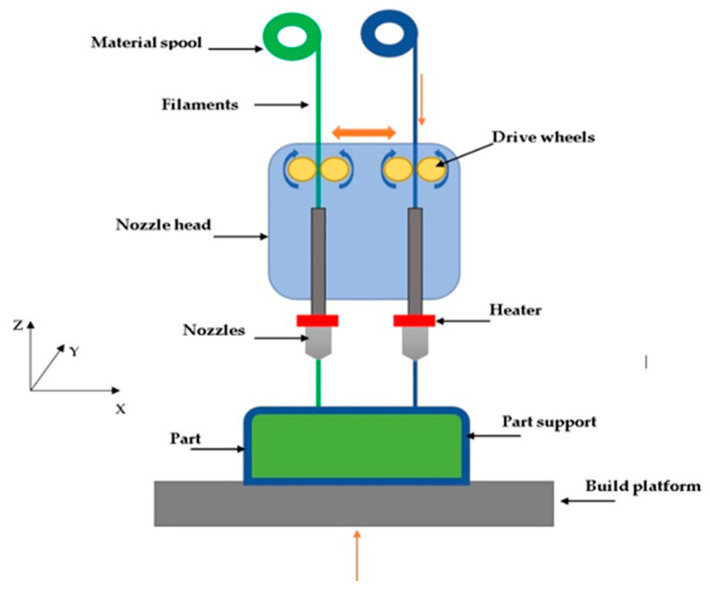
Schematic of FDM components.

FDM was invented and developed by S. Scott Crump (co-founder of Stratasys) in 1988 [21]. This process is user friendly and is available from desktop to large size depending on the requirement. Machine cost is started from less than $1000 to $20,000. This technology consists of different components as shown in Figure 1 [22]. Stepper motors, filament material, nozzle(s), and heater are the main components of FDM. The most effective part is the extruder (nozzle). The machine can have one or two extruders based on needs. Usually, the second nozzle is used to print the support structure.

The printing process is started by slicing the design and convert the file to STL format. Stepper motors start to push through the solid material which is in the form of a filament spool. Gears inside the nozzle head push the material and the extruder melts the material at melting point temperature and deposits molten material on the bed. The material starts to solidify and bond to each other. This procedure is repeated for each layer until the end when the sample is completed [23]. As mentioned support structure is also needed in specific angles to avoid the material drops and tighten gaps to reach high accuracy [24]. After printing this structure should be removed to enhance surface quality and mechanical properties. Post-processing techniques can be useful to eliminate support or improve product quality [25]. There have been studies to eliminate this issue by developing multiaxis or jointed-arm robot FDM [26,27]. However, some obstacles still exist in these developments like collisions and difficulties to control the process. Machining [28], chemical process [29], laser cladding process [30], and hot air jet [31] are professional post-processing techniques that have been used to enhance the part’s quality. Plastics are the main materials in this process and are available in different colors. The most common materials are Acrylonitrile butadiene styrene (ABS), Polylactic acid (PLA), Polypropylene (PP), Polycarbonate (PC), Nylon, and Thermoplastic polyurethane (TPU) [32].

**Figure 2:**
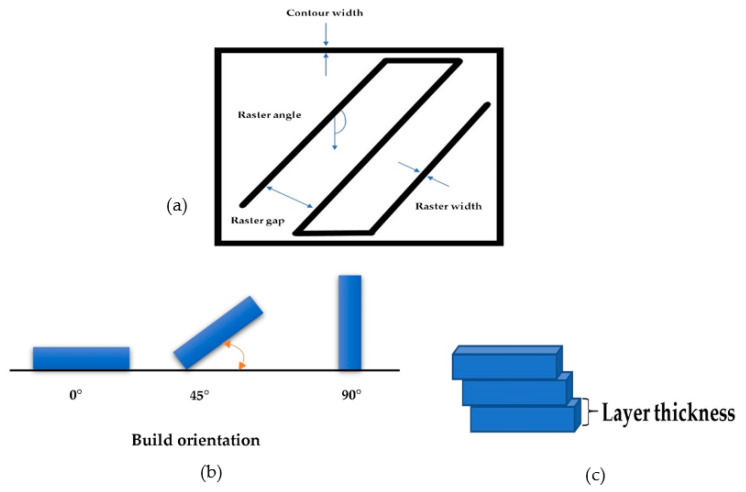
(**a**) FDM path parameters, (**b**) build orientations, and (**c**) layer thickness.

Additionally, polymer composites, ceramic slurry, food pastes, and biological pastes can be printed by FDM based on requirements. As an example, Matsuzaki et al. [33] printed continuous fiber composite (Jute) by nozzle impegration technique. The advantage of FDM process over other processes is that the material is not as toxic as the Stereolithography (SLA) process. Thus, materials are used widely to print toys and various products. A wide range of parameters affects mechanical properties of final products in FDM. Build orientation, layer thickness, nozzle temperature, nozzle diameter, bed temperature, infill pattern, infill density, and printing speed are the most critical elements to meet high accuracy [34]. As shown in Figure 2 [35], raster angle, air gap, raster width, and wire-width are other elements that affect surface quality, mechanical properties [36,37]. Undoubtedly, FDM is a capable process to produce samples faster with lesser cost but there have been some issues in this process that should be considered. Printing layer by layer leads to staircase defects in the final sample and mechanical properties and surface texture are affected [38]. Furthermore, warping and shrinkage are other obstacles in FDM due to non-optimized process parameters or poor temperature [39]. On the other hand, thermoplastic properties are hard to identify due to large deformation and tend to have less stiffness and strength compared to metal [40]. In addition, the quality of surface roughness is not as good as other processes such as SLM [41]. Achieving tight tolerances is difficult in FDM and the dimensional accuracy is around ±0.2 mm and ±0.5 mm which is not as tight as machining process tolerance. Therefore, smooth surface texture is hard to achieve, and using post-processing techniques is a way to improve quality of 3D printed products. Furthermore, engineering plastics are divided into isotropic and anisotropic which finding properties such as elongation at break, young modulus, and tensile strength are vital to achieve accurate results [42]. Ziemian et al. [43] found out the anisotropic behavior of ABS400 material in FDM process by tension test. In this study, 0° raster orientation showed the largest ultimate and yielded strength which was 93% of injection-molded specimen [44].

Many types of research have been conducted on process parameters to find out the behavior of FDM in various conditions and eliminate process issues [45,46]. As an example, Yang et al. [47] investigated five process parameters to find out their influence on printing time, surface roughness, and tensile strength. Analysis of variance (ANOVA) was used to examine their effects and it was founded nozzle diameter and layer thickness had the most impact on surface roughness and tensile strength. Further, printing plane is the most valuable factor affects printing time and part thickness [48]. Build orientation and layer thickness were analyzed by [49] with an experimental and theoretical model to investigate the tensile strength of 3D printed PLA products. In-layer failures with their separation angle in each plane were examined to find out the capability of the theoretical model to predict the tensile failures. Besides, different nozzle diameter from 0.2 mm to 0.8 mm and is the heart of FDM process [50]. Nozzle temperature can be modified from 60 °C to 280 °C depends on material melting point. by increasing the nozzle diameter and layer thickness, the printing time is increased as well. It should be noted build orientation is an important factor in FDM due to its effects on surface texture, dimensional accuracy, and material consumption [51]. Sukindar et al. [52] examined nozzle diameter by analyzing different extruder angles and diameter in experimental and Finite element analysis (FEA) process to investigate the pressure drop and its influence on product quality. Another issue in FDM printed samples is low strength which is caused by poor layer binding. non-optimized temperature or parameters lead to poor adhesion between layers. Build orientation is the next important factor that starts from 0° to 180° refers to the product’s design [53]. Perpendicular angles need more support structure compared to the horizontal and vertical orientations and printing time is longer when the machine uses supports. Buj-Corral et al. [54] found out the surface roughness values (e.g., skewness (S_ku_), kurtosis (R_ku_), and maximum height of profile (R_z_)) increased by increasing build orientations from 5° to 85°.

Meanwhile, the machine follows different infill patterns from linear to cubic, and samples can be hollow or solid based on infill density which starts from 0% to 100%. Alsoufi et al. [55] analyzed the surface roughness of the inner and outer face of PLA+ thermoplastic samples with 0% infill density. Infill pattern has a great effect on mechanical properties and flexural strength [56]. Moreover, lightweight products are the main goal of industries while reducing weight leads to poor strength and low mechanical properties. By choosing the optimum infill pattern and infill percentage, it is possible to reach the highest strength while material consumption is lowest [57]. It should be noted by increasing infill density, tensile strength, flexural strength, and mechanical properties are increased as well [58,59]. This means a high correlation between mechanical properties and infill parameters which are discussed in this study. Thus, by finding optimum infill pattern and density, the highest properties are achieved without wasting material. Ćwikła et al. [60] worked on infill density, infill pattern, shell thickness, and printing temperature to analyze their influence on tensile strength in ABS 3D printed dog-bone samples. Infill pattern was the most effective parameter in products’ resistance and samples weight. It was founded that honeycomb pattern showed higher mechanical resistance compared to other infill patterns [61]. Furthermore, Akhoundi et al. [62] worked on infill pattern and infill percentage of 3D printed PLA products to find out the behavior of concentric, rectilinear, hilbert curve, and honeycomb patterns in binding and adhesion after printing and their effects on the product’s strength. A novel study has been conducted to investigate the weight, stiffness, and peak load by Podroužek et al. [63] on bio-inspired 3D infill pattern. Light-weight complex cylindrical structures include Gyroid, Schwarz D, and Schwarz P were printed and analyzed by FEA (ANSYS^®^ Mechanical^TM^). Slicing strategy and build orientation were also conducted on a dog bone and differences were found out based on Coefficient of variation (COV). This technique is useful when build orientation is not aligned with extrusion angle [64]. ABSplus (p430) and ABS 3D printed dog bone samples were examined by Lee et al. [65] to determine the ultimate stress (σ_u_) in various build orientations. ABSplus (p430) material showed higher tensile stress while strain energy of materials depended on build angle. Therefore, build orientation is highly effective to maximize strength and stiffness of 3D printed products. Dave et al. [66] compared concentric, rectilinear, and hilbert curve pattern with 60%, 80%, and 100% infill density in PLA material and found out concentric with flat part orientation had the highest values of tensile strength which was 37.7 MPa. Another study showed printing patterns behavior was similar but less than 5% variation in maximum tensile strength was recorded [67]. Moreover, Gopsill et al. [68] analyzed beam shaped samples with honeycomb infill under four loading scenarios by using FEA. This technique is helpful for design optimization and reaching the highest strength with optimum infill density for complex products. In another study, Hanon et al. [69] examined the strength of PLA and Polyethylene terephthalate-glycol (PETG) and made a comparison in different orientations from +45° to −45° with straight and honeycomb pattern. PETG indicated better elongation results, and Y orientation with 0° raster direction showed the highest tensile strength value. Flexural rigidity and infill parameters were investigated by three-point bending, free vibration, and buckling tests. It was found that flexural rigidity dropped below 10% infill. Samykano et al. [70] developed a mathematical model to analyse FDM parameters. They found 80% infill, 0.5 mm layer thickness, and 65° rastle angle were the optimum parameters for ABS material.

In brief, by controlling and choosing proper process parameters, reaching high quality and required strength are achievable. Studies showed how it was possible to maximize the strength of 3D printed samples without sacrificing mechanical properties. Nevertheless, there have not been researches on complex infill pattern and a combination of various patterns in 3D printed PLA product. Hence, this study tends to investigate the effects of 3D complex shape patterns in FDM printed products and find out the capability of FDM in printing combined patterns. This goal was done by an association of layers with different features. Each layer had exclusive pattern which was bound to each other. This study also can be used as a novel technique in different AM technologies for lattice and sandwich structures due to the combination of infill patterns [71,72]. The effects of build orientation on strength are also analyzed in combined pattern dog-bone shape samples. The strength and mechanical properties of specimens were analyzed to find out the printing defects and capabilities. FEA was used to investigate the layer’s behavior under tensile test. After combining the pattern, the sample was examined in Abaqus^®^ to find out the defects and deformation.

## 2. Materials and Methods

**Figure 3:**
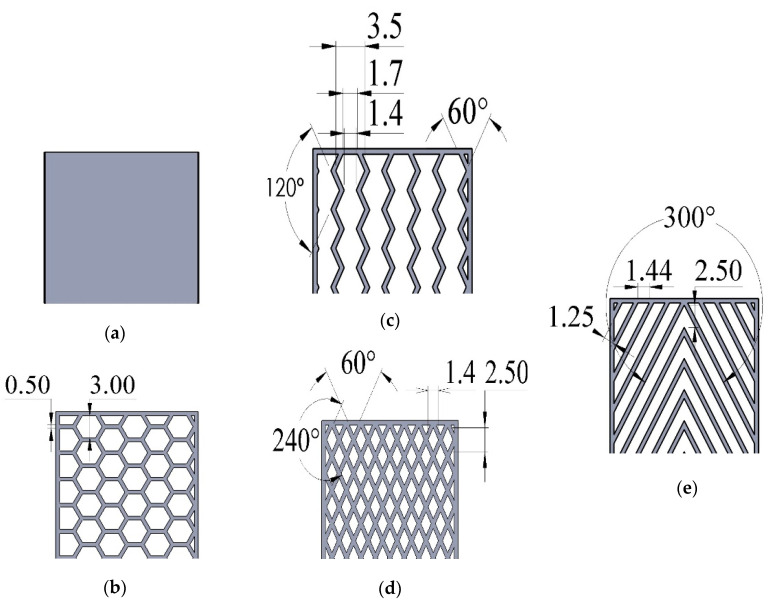
(**a**) Solid, (**b**) honeycomb, (**c**) wiggle, (**d**) grid, and (**e**) rectilinear patterns.

**Figure 4:**
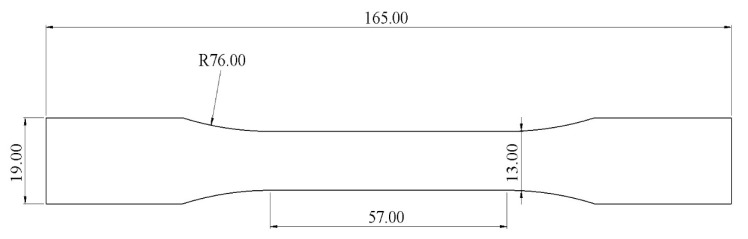
Computer aided design (CAD) design of dog-bone shaped sample.

As mentioned in the literature, each infill pattern has specific features and characteristics. Various researches showed by choosing a proper pattern and optimum build orientation, the highest strength and stiffness are achievable. Besides, material properties have effects on surface texture and strength of final products. Various patterns like honeycomb, rectilinear, grid, cubic, hilbert, and others have been investigated to analyze their effects. Building time, energy consumption, material usage, strength, and surface quality are the main criteria when patterns are changed. However, analyzing a product that has different patterns in each layer has not been conducted yet. Hence, in this paper tensile strength of 3D printed products was analyzed based on ISO/ASTM D638 (standard test method for tensile properties of plastics). The machine is not capable to print a sample with different patterns. Thus, to find out the best results, five patterns were chosen to be designed in Solidworks^®^. As shown in Figure 3, solid, honeycomb, wiggle, grid, and rectilinear were chosen due to the high strength properties that discussed before. Details of each layer are provided in Figure 3 as well. The chosen infill density was 70% to design the patterns and show the details properly. Due to layer by layer adhesion, these designs were picked to minimize the distances because of different features. In addition, 0.5 mm thickness was chosen to design patterns because at 0.4 mm thickness, the slicer could not detect the infill patterns properly. The dog bone sample was designed based on tensile strength standards. Type 1 was chosen with 7 mm thickness and Figure 4 shows the dimensions of the specimen.

**Figure 5:**
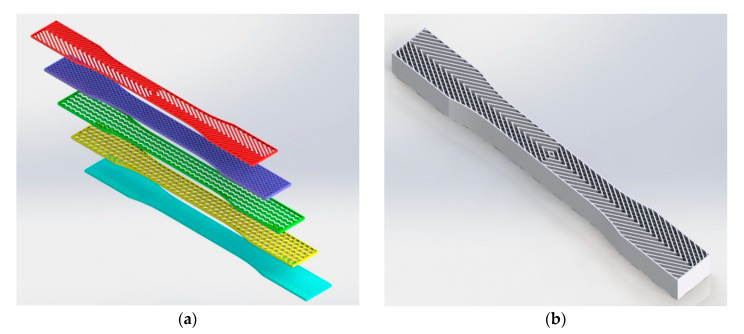
(**a**) Classified layers and (**b**) CAD model according to ISO/ASTM.

The first and the last pattern had 1.25 mm thickness while others were 1.5 mm, respectively. Each layer was designed separately and all of them were combined to make the final product for the printing process. Figure 5a,b show the classification of each layer in the dog bone product and the final design of the sample which was used to be printed by FDM machine. The order of the layer was started with solid, honeycomb, wiggle, grid, and rectilinear. It should be noted that designing layers is important to avoid gaps and voids due to the different patterns. Thus, combining patterns should be done by proper assembling that each pattern attaches to the previous one.

On the other hand, FEA is a powerful technique to examine properties and product’s behavior under loads and tensions [73]. Finding the sample’s reaction was conducted by finite analysis due to its capability to examine various geometries. Hence, finding the behavior of each pattern is necessary to determine the defects in this case. As discussed in the literature, patterns showed different characteristics and properties. These five patterns were analyzed in Abaqus^®^ (2020 version, Dassault Systèmes, Vélizy-Villacoublay, France) to examined the samples’ differences and strengths separately. To conduct accurate results, 1.5 mm thickness was assigned for all 5 layers, respectively. The samples were imported from Solidworks^®^ (2020 version, Dassault Systemes) due to the complex design and were considered as isotropic parts. This method helps to find out, in the final sample, which pattern has the highest strength while the weight is the lowest. Furthermore, crack points and failures are identified in the final specimens. Static analysis was done on all patterns with constant parameters to find out displacements and strengths. System International (SI) in millimeter (mm) unit was used for Abaqus^®^ analysis. PLA material properties from the Polymaker catalog was assigned for the simulation process due to its diversity and good mechanical properties. Furthermore, specimen weights were recorded to identify the weight differences. Quadratic tetrahedral elements of type C3D10 mesh was assigned for each sample to receive accurate results among all samples. Table 1 shows the details of each pattern and assembled specimen, respectively.

Moreover, loads and fixtures were assigned both sides of the samples refer to ISO/ASTM tensile test analysis. A concentrated force with 1000 N value was chosen for one side and the other side was fixed by Symmetry/Encastre fixture to proceed with the analysis properly. Deflections and displacements were recorded to determine the defects and failure in each pattern and final sample. Hence, a comparison between the final sample and 3D printed products to find out the failure and cracks in various build orientations.

The printing process was done by Ultimaker 2+ machine (Ultimaker, Geldermalsen, Netherlands) which was capable to print various materials from thermoplastic to composite. PLA material with white color from PolyLite^TM^ Polymaker was chosen for the printing process. PLA is a biodegradable material that is highly used in manufacturing companies. Material properties are provided based on the supplier catalog in Table 2. As shown in Table 1, the properties of PLA material are higher while the melting point is less than ABS material.

**Figure 6:**
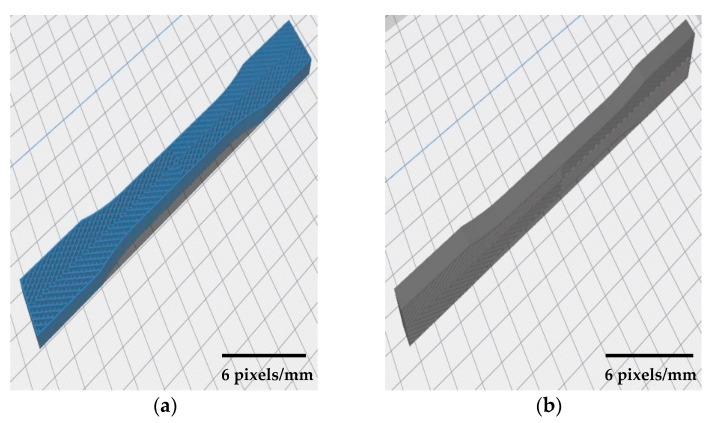
Schematic of sample orientation in (**a**) flat side and (**b**) on-edge side.

**Figure 7:**
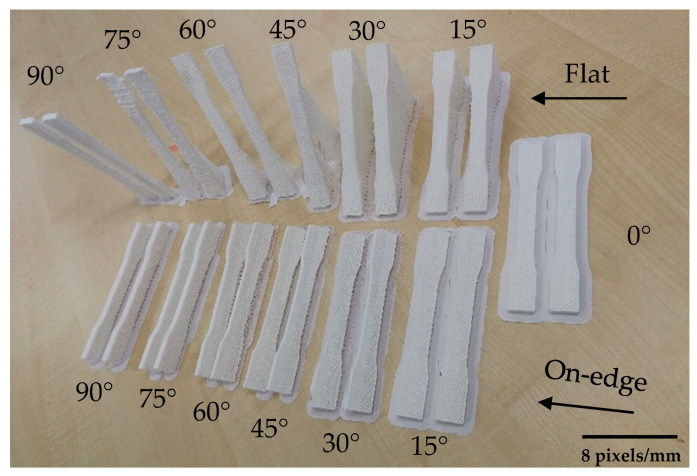
A set of 3D printed PLA samples.

The samples were printed in flat and on-edge side in different build orientations (see Figure 6). Samples were printed by following these two sides to determine the effect of combined patterns on the product’s strength. CuraEngine^®^ software (4.5 version, Ultimaker, Geldermalsen, Netherlands) was used to slice the CAD design and prepare the printing parameters which were constant for all specimens, as shown in Table 3. Solid infill (100%) was chosen to determine the patterns that were designed and the following parameters are conducted from the supplier’s catalog. A total of 42 samples were printed by FDM machine for each orientation and a set of samples is shown in Figure 7. In perpendicular samples, the machine used support structure to avoid the material drops. Thus, this may affect the surface texture and mechanical properties of final products.

The following step was to analyze the strength of each sample. INSTRON 3365 machine (Instron, Norwood, Massachusetts, United States) with 1000 mm/min maximum speed was used to measure tensile properties. This tensometer was a vertical machine with 5 kN capacity. Samples were put between the jaws and fixed by grippers. Deformation speed of 5 mm/min was applied at room temperature via software to continue the progress. The final results were analyzed to find out the maximum strength of samples in different build orientations.

## 3. Results and Discussion

As discussed in the literature, many elements are effective to increase stiffness and strength. Material properties, FDM parameter, layer binding, infill pattern, and build orientation are the important elements that have effects on deformations and deflections. One obstacle in this research was how to design patterns to bind them properly without gaps and voids between each layer. Hence, the first goal was to analyze layer binding to determine the defects and errors. The reason why the patterns should be designed in CAD software is that the slicing softwares are not able to slice a product and assigned different patterns in a product. Therefore, developing a new algorithm to combine patterns in different layers in the slicing process helps to conduct products with higher strength and stiffness. Analyzing the pattern’s strength by using FEA showed how they react when loads were applied.

The strength of each pattern in dog-bone shape was recorded to make a comparison on weight differences and their strength. Von mises and displacements were analyzed under the same condition to determine how patterns react under loads. Maximum tension was also examined in each pattern to find out their behavior. One element was chosen to analyze stress-strain of each sample from solid to the rectilinear pattern, respectively. As shown in Table 4, Von mises of each layer were recorded to show the deflection and displacements. The maximum and minimum of Von mises for each pattern are visible as a legend in Table 4. A comparison between each pattern was conducted to find out which one was the strongest by reducing the weight from solid sample. Thus, the Von mises analysis showed the pattern behavior under tensile loads. Undoubtedly, the solid pattern had the highest strength among patterns but finding the most durable pattern with lower weight was the main point.

**Figure 8:**
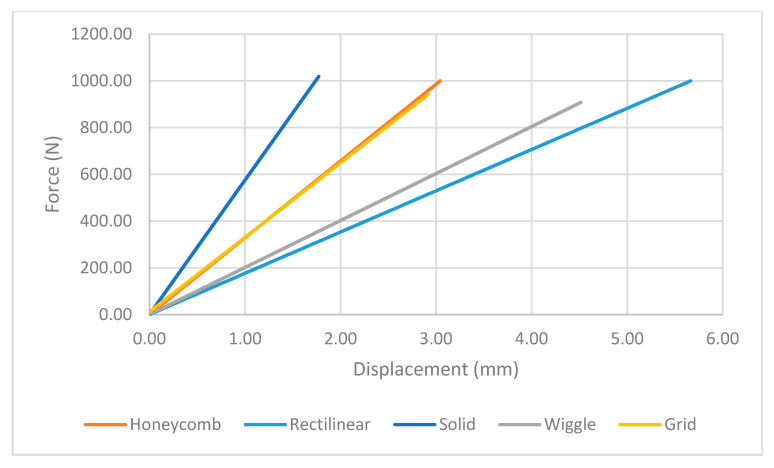
Force-displacement line chart for patterns.

At the assigned node, it was found out the maximum stress happened for the rectilinear and wiggle pattern and these patterns had the weakest strength. Besides, the grid and honeycomb pattern showed the highest strength compared to the solid sample. This means by using grid and honeycomb patterns, printing strong product is achievable while the weight is minimized. It should be noted, honeycomb had a lower weight with 1.39 g than grid pattern with 1.68 g. To determine the optimum pattern, force-displacement was also analyzed for each pattern to find accurate results. Hence, Figure 8 shows the displacements of patterns under 1000 N tensile load. The rectilinear pattern had the largest value in stress–strain value while the solid sample showed the highest durability and lowest value of stress. Force-displacement diagrams were analyzed to identify the displacements of patterns under tensile loads. As shown in Figure 8, the rectilinear pattern has the highest displacement at 1000 N while the lowest one is for solid. The displacement of honeycomb and grid sample were almost the same around 3 mm while honeycomb was lighter compared to grid pattern. In brief, by comparing patterns, it was found out grid and honeycomb had the highest strength compared to other patterns based on stress, displacement, and weight [61]. Moreover, 3D printed samples were analyzed to find out the printing capability and the best orientation to print a product with different infill patterns.

**Figure 9:**
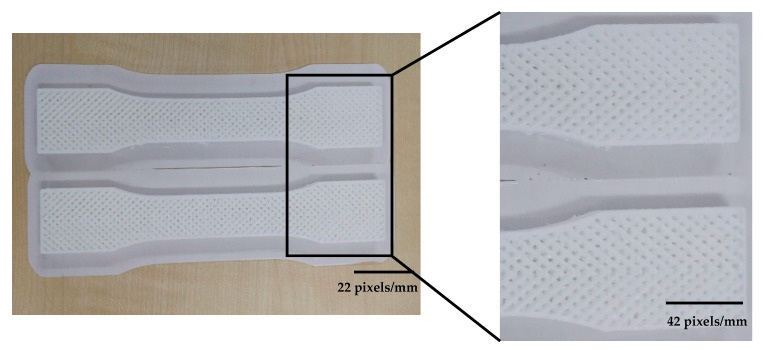
Quality of sample in 0° orientation.

**Figure 10:**
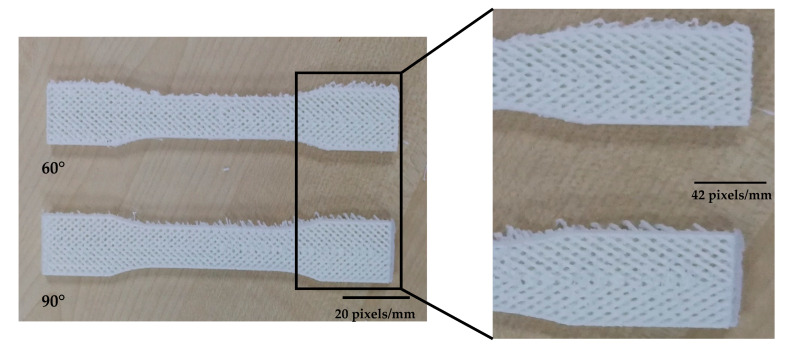
The quality of 60° and 90° on-edge samples.

**Figure 11:**
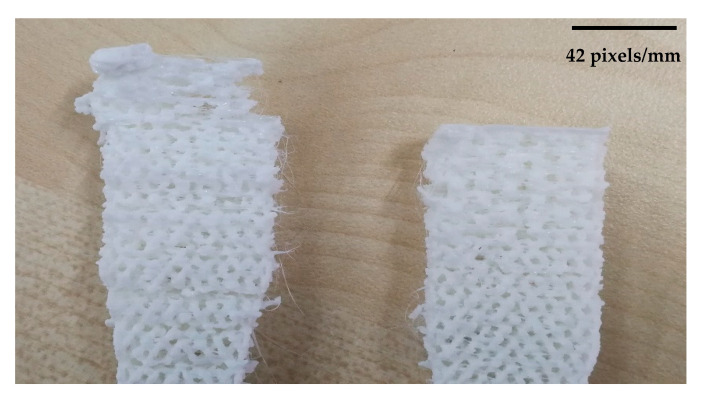
Defect in 75° flat samples.

These patterns were chosen to attach the lines and sluts effectively. However, the machine used dot supports in some areas to tighten the gaps while supports were small and due to the small size, they were not effective in whole process. Figure 9 shows the patterns are situated layer by layer properly. The quality of the printed pattern at 0° angle was good and showed the FDM process is capable to combine patterns but these patterns must be designed in CAD software separately due to the system limitations to combine patterns. However, by increasing the build orientation the quality of layers became worse in both flat and on-edge. As shown in Figure 10, the quality of 60° and 90° was not as good as 0°. Therefore, printing combined pattern needs to be done with alignment through the printing direction. An issue happened in 75° orientation sample in flat parts. The quality of part was like other perpendicular sample but the main problem was at the end of the printing process the machine could not adhere the layers properly and the material dropped or the binding was not good at all (see Figure 11). Therefore, this angle was removed due to this limitation, which might happen because of poor printing speed.

**Figure 12:**
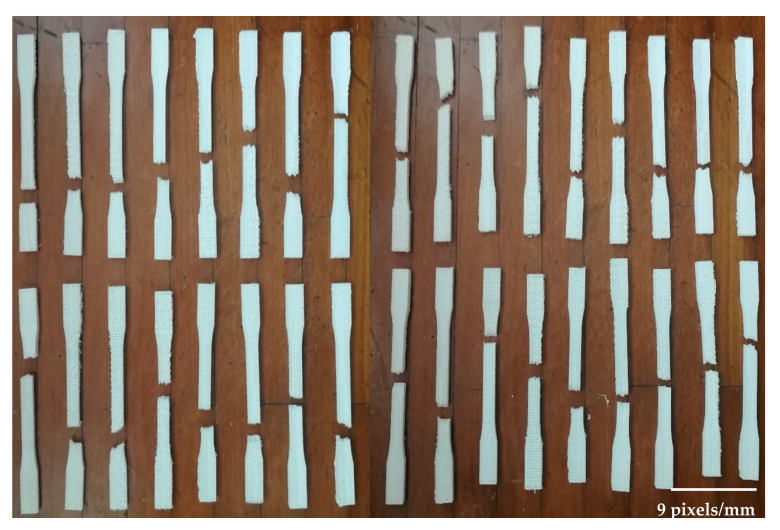
Failure in random specimens.

As shown in Figure 12, fatigue and crack in random samples are recorded to show the fatigue’s line and the exact place that the parts cracked. The reason that samples were crack at different points was due to the effects of build orientation on layer adhesion. This means by changing build angle, the strength between each layer becomes weaker and it leads to different results. Besides, samples were printed in two directions which had a great effect on mechanical properties. All samples were analyzed one by one to determine the strength of each orientation under tensile load. Three specimens were analyzed in each angle and the final loads were recorded accordingly. Therefore, the average maximum tensile load for flat samples is recorded in Table 5 to clarify the differences. Meanwhile, Table 6 shows the differences in maximum tensile load in on-edge printed specimens. As shown in Table 5, by increasing the build orientation the strength and stiffness of printed specimens became weak. The average value for forces and extensions of three samples was conducted by the trendline equation for 3 graphs. In flat specimens, the strongest sample was 0° while the weakest was 60° with the average value of 1427.68 N and 570.77 N with the displacement of 11.34 mm and 5.13 mm, respectively. Due to the defect that was mentioned before, 75° flat samples were not investigated. Moreover, the same results happened for on-edge parts under tensile loads. This means, by increasing the build orientation, the stiffness becomes weak. The highest value was recorded for 0° with 1466.23 N and the lowest one belonged to 75°. These results happened due to the poor layer adhesion and also the differences in infill patterns for each angle. On the other hand, after 60° and 75° angle, higher loads were needed to break the samples. In 90° angle, for both flat and on-edge sample the value of fatigue was higher compared to 60° and 75°. In brief, by printing in horizontal direction better adhesion and stronger samples can be achieved [66]. Meanwhile, by looking at the results, it can be concluded on-edge parts were stronger compared to flat ones. For example, 15° samples in flat printing cracked at 890.29 N while they cracked at 1332.67 N in on-edge samples. To examine the tensile process, stress–strain charts for both flat and on-edge parts are provided to determine the effects of combining infill patterns on mechanical properties.

**Figure 13:**
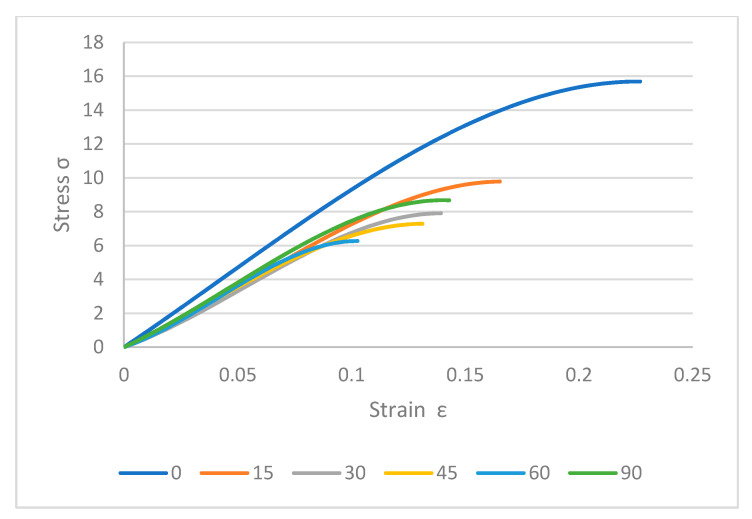
Stress–strain curves for flat samples.

**Figure 14:**
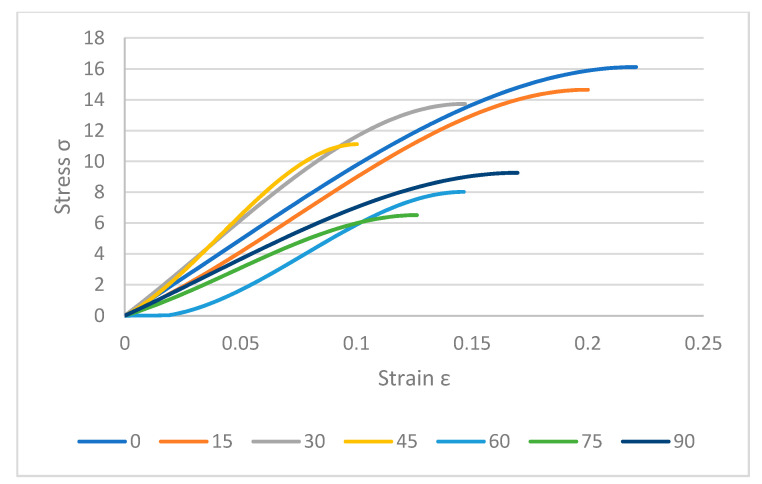
Stress–strain curves for on-edge samples.

Figure 13 shows the variety of loads that were needed to crack specimens. The maximum stress was recorded for 0° which was around 16 MPa while it was less than 10 MPa for others. Thus, by increasing the build angle, the layers adhere poorly so a lower load was needed to break the samples. Meanwhile, the same thing happened for on-edge samples but they showed better results compared to flat samples in perpendicular ones. Samples required higher loads to crack completely and were stronger in layer binding. As shown in Figure 14, 15° specimen needs more than 14 MPa which is close to 0° angle. Besides, other on-edge samples were in better condition in stiffness and strength. The worst sample in flat samples was 60° and for on-edge specimens was 75° angle. In brief, the best quality and properties were achieved for 0° angle. The best quality of samples was good at 0° in flat printing [45]. However, printing complex and combined patterns can be done at this angle, the 3D printers are not capable of combining different patterns in a product.

**Figure 15:**
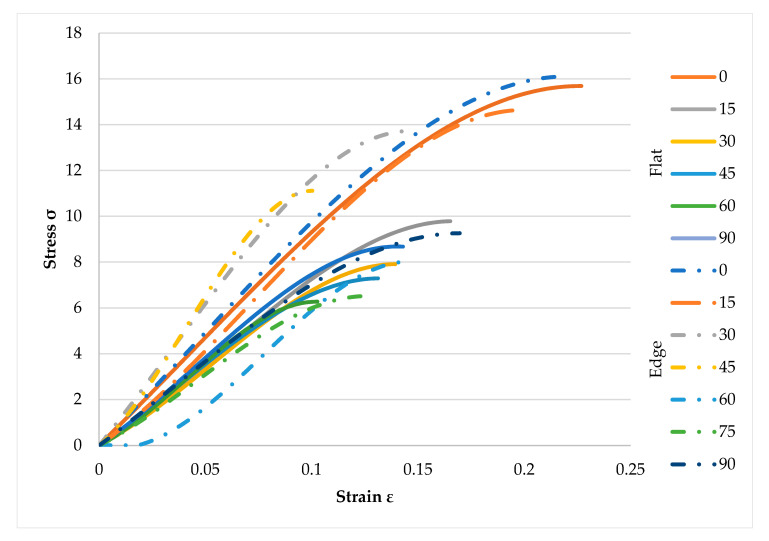
Stress-strain for both flat and on-edge fabricates.

To show the differences between two directions, Figure 15 is provided. As shown in Figure 15, on-edge samples had higher stress value. These specimens were stronger compared to the flat samples due to the printing angle. As an example, 0° samples in flat direction with the value of 15.6 MPa were weaker compared to the on-edge samples with the value of 16.2 MPa. Moreover, results showed the same direction for the other specimens in various orientations. Furthermore, 75° samples in on-edge fabricates were stronger than 60° specimens with an average value of 6.3 MPa and 6.1 MPa, respectively. This means on-edge samples had higher strength and required load to crack samples should be higher compared to the flat ones.

## 4. Conclusions

The 3D printing processes are widely used in different applications due to their high capability to print various materials with different properties. FDM is one of the most common technologies that can be used to print thermoplastic and composite materials. Many parameters affect surface quality and mechanical properties. Layer thickness, build orientation, infill pattern, infill density, nozzle temperature, and bed temperature are the main elements in this process. This study investigated combining different patterns in a product and examined their effects on mechanical properties in various build orientation. It was found the FDM process was not capable to combine patterns by itself. Therefore, if the patterns are designed by CAD software, it is possible to print a product with various infill patterns. Results showed infill pattern and build orientation had drastic effects on mechanical properties. Solid, honeycomb, wiggle, grid, and rectilinear were combined in a dog-bone shaped sample. Using FEA showed honeycomb and grid patterns were strongest among other patterns while they had lower weight. On-edge printed samples had better mechanical properties compared to the flat specimens. In both directions, the 0° sample had the highest strength and the best quality compared to vertical and perpendicular specimens. Meanwhile, the printer could not print the 75° angle in flat direction. Furthermore, 60° and 75° orientations had the weakest adhesion among all samples. This technique can be used for future researches on machine and software development to combine different patterns in slicing software. Combining various patterns can be used in different applications to achieve stronger products without sacrificing properties. By developing a novel software or technique to eliminate this limitation, printing a highly complex pattern shape is possible.

## Figures and Tables

**Table 1:** Samples′ features in FEA according to PLA material properties.

Pattern	Solid	Honeycomb	Wiggle	Grid	Rectilinear
**Material**	PLA	PLA	PLA	PLA	PLA
**Mesh size**	2	2	2	2	2
**Force (N)**	1000	1000	1000	1000	1000
**No. of nodes**	12,929	25,448	13,744	47,142	18,024
**No. of elements**	7133	11,932	5050	22,469	6932
**Mass (g)**	4.46	1.39	1.09	1.68	1.49
**3D mesh design**	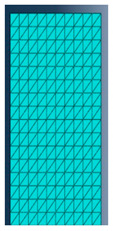	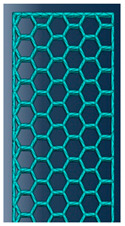	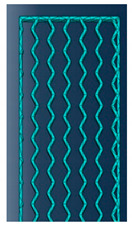	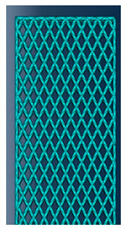	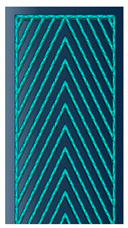

**Table 2:** Material properties.

Material	PLA
Density (g/cm^3^)	1.17
Printing temperature (°C)	190–230
Printing speed (mm/s)	40–60
Bed temperature (°C)	25–60
Tensile strength (MPa)	46.6 ± 0.9
Young’s Modulus (MPa)	2636 ± 330

**Table 3:** Printing parameters.

Side	Flat and on-Edge						
**Build orientation (degree)**	0	15	30	45	60	75	90
**Nozzle diameter (mm)**	0.4	0.4	0.4	0.4	0.4	0.4	0.4
**Filament diameter (mm)**	2.85	2.85	2.85	2.85	2.85	2.85	2.85
**Layer thickness (mm)**	0.15	0.15	0.15	0.15	0.15	0.15	0.15
**Infill density (%)**	100	100	100	100	100	100	100
**Printing pattern**	Linear	Linear	Linear	Linear	Linear	Linear	Linear
**Nozzle temp. (°C)**	200	200	200	200	200	200	200
**Bed temp. (°C)**	60	60	60	60	60	60	60

**Table 4:** Schematic of Von Mises value and stress.

Pattern	Von Mises (MPa)
Solid	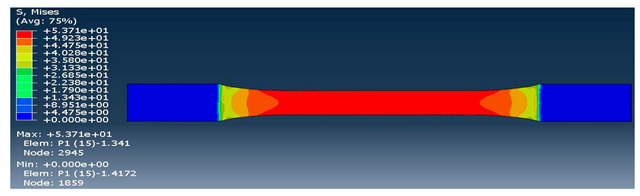
Honeycomb	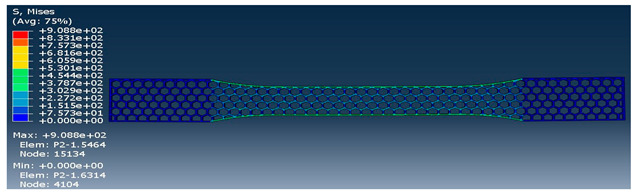
Wiggle	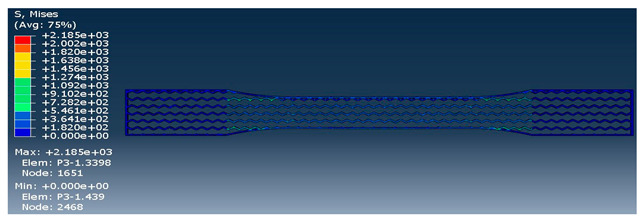
Grid	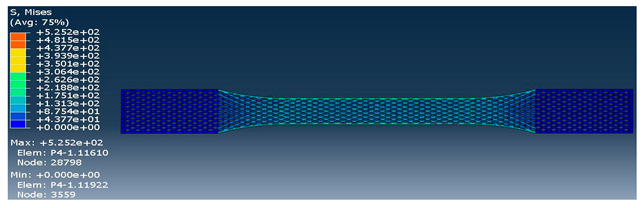
Rectilinear	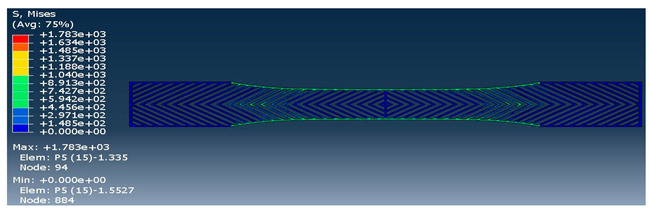

**Table 5:** Maximum tensile load for flat samples.

Build Orientation	Sample	Force (N)	Extension (mm)	Average Force (N)	Average Extension (mm)
0°	1	1425.88	11.33	1427.68	11.34
	2	1375.37	11.8		
	3	1493.72	10.8		
15°	1	903.36	9.52	890.29	8.26
	2	821.03	9		
	3	836.80	9.49		
30°	1	745.33	6.76	719.71	6.97
	2	696.23	7.48		
	3	719.47	7.2		
45°	1	637.55	6.12	663.30	6.56
	2	752.17	7.7		
	3	640.49	6.4		
60°	1	582.28	4.95	570.77	5.13
	2	628.30	5.95		
	3	583.87	5.2		
75°	1	Not Available	Not Available	Not Available	Not Available
	2	Not Available	Not Available		
	3	Not Available	Not Available		
90°	1	825.54	6.48	789.66	6.9
	2	727.75	6.9		
	3	822.49	7.25		

**Table 6:** Maximum tensile load for on-edge samples.

Build Orientation	Sample	Force (N)	Extension (mm)	Average Force (N)	Average Extension (mm)
0°	1	1671.08	11.06	1466.23	11.04
	2	1439.46	13.32		
	3	1404.56	9.8		
15°	1	1327.81	10.08	1332.67	10
	2	1360.13	9.88		
	3	1311.18	10.03		
30°	1	1275.36	7.5	1249.17	7.35
	2	1220.57	7.36		
	3	1253.38	7.2		
45°	1	1094.31	4.62	1011.54	5.01
	2	1007.40	5.5		
	3	981.44	5.28		
60°	1	638.32	7.59	730.23	7.32
	2	979.27	4.68		
	3	776.659	9.75		
75°	1	604.26	6.3	593.17	6.31
	2	693.25	7.08		
	3	511.27	5.78		
90°	1	804.23	10.54	842.76	8.48
	2	856.08	7.84		
	3	933.35	8.1		
